# Transport-injury burden among children and adolescents in the Western Pacific Region, 2000–2023, with projections to 2035

**DOI:** 10.3389/fped.2026.1946652

**Published:** 2026-10-14

**Authors:** Rongxiang Sun, Qinglong Jian, Chen Ye, Quanneng Luo, Kenyou Zhou, Huilong Guo, Guohua Wang, Chuncheung Chan

**Affiliations:** 1 Department of Emergency and Disaster Medicine Center, the Seventh Affiliated Hospital, Sun Yat-sen University, Shenzhen, China; 2 University Center of Orthopaedic, Trauma and Plastic Surgery, University Hospital Carl Gustav Carus at TUD Dresden University of Technology, Dresden, Germany; 3 Department of Orthopedic Medical Center, Hunan Provincial People’s Hospital, The First Affiliated Hospital of Hunan Normal University, Changsha, China; 4 Department of Spine, the Seventh Affiliated Hospital, Sun Yat-sen University, Shenzhen, China

**Keywords:** children and adolescents, decomposition analysis, forecasting, global burden of disease, health inequality, transport injuries, Western Pacific Region

## Abstract

**Background:**

Transport injuries remain a major preventable cause of death and disability among children and adolescents. Evidence on long-term trends, geographic inequalities, and future burden across the Western Pacific Region (WPR) remains limited. We assessed transport-injury burden among people aged <20 years in the WPR from 2000 to 2023 and projected trends to 2035.

**Methods:**

We analysed Global Burden of Diseases, Injuries, and Risk Factors Study 2023 estimates for 33 WPR countries and territories. We assessed incidence, prevalence, deaths, and disability-adjusted life years (DALYs) from 2000 to 2023. We mapped 2023 age-standardised rates, examined four age groups by sex, and decomposed changes into demographic and epidemiological components. Sex-specific autoregressive integrated moving average (ARIMA) models were used to project age-standardised outcomes from 2024 to 2035.

**Results:**

Incident cases decreased from 5,034,407 [95% uncertainty interval (UI), 4,180,502–5,955,077] in 2000 to 2,433,402 (1,988,777–2,920,981) in 2023. The age-standardised incidence rate fell from 812.69 to 428.74 per 100,000. The annual percentage change was −2.82% (95% confidence interval, −3.04 to −2.61; *P* < 0.001). In 2023, incidence varied 4.48-fold and mortality varied 38.2-fold across locations. Males aged 15–19 years had higher incidence, mortality, and DALY rates than females of the same age. Epidemiological change drove most of the regional reduction. Between 2024 and 2035, ARIMA models projected male age-standardised incidence and prevalence rates to decline modestly, while female rates remained nearly stable. Mortality and DALY projections crossed zero and were excluded from quantitative interpretation.

**Conclusions:**

The transport-injury burden among children and adolescents in the WPR decreased substantially, but marked geographic and age-sex inequalities persisted. The 2024–2035 projections suggest that non-fatal burden may decline only modestly, with little change among females. Prevention should prioritise older adolescent males and high-mortality settings, while longer-term policy planning requires more robust forecasting.

## Introduction

Transport injuries are an important source of preventable death and disability among children and adolescents in the Western Pacific Region (WPR). Burden increases across adolescence and is consistently higher in males (1–4). Although age-standardised mortality and disability-adjusted life-year (DALY) rates from road injuries have fallen in many settings, progress varies, and large numbers of events may persist despite lower rates (5–7). For planning, rates indicate burden relative to population size, whereas counts more directly reflect demands on prevention, emergency care and rehabilitation.

Existing evidence from the WPR is difficult to compare across studies. Research from China has examined mortality trends and age–period–cohort patterns (8), whereas analyses of low- and middle-income countries and Pacific island countries have used different populations and outcomes (9, 10). Studies in Malaysia and China have focused on specific national or age-defined populations (11, 12), and regional policy research has documented continuing gaps in protection for children and adolescents (13). No study has compared incidence, prevalence, mortality and DALYs within a common framework covering the current WPR membership. Location-specific rates have also rarely been examined alongside event counts, despite the large differences in population size across the region.

Available studies do not show whether lower transport-injury rates across the WPR have translated into fewer incident and prevalent cases, deaths and DALYs. They also do not establish whether the locations and age–sex groups with the highest rates account for the largest numbers. Examining these relationships requires fatal and non-fatal outcomes to be assessed over the same period and geographic scope.

Using Global Burden of Diseases, Injuries, and Risk Factors Study (GBD) 2023 estimates, we examined transport-injury burden among people aged <20 years in 33 WPR countries and territories from 2000 to 2023 (14, 15). Our objectives were to quantify regional temporal trends; compare 2023 incidence, prevalence, mortality and DALY estimates across locations; and describe age- and sex-specific patterns. We interpreted rates and counts together to distinguish populations with high estimated rates from locations facing the largest service caseloads.

## Materials and methods

### Study design and data sources

GBD data were obtained from the publicly available Global Burden of Disease Study 2023 Results Tool maintained by the Institute for Health Metrics and Evaluation (IHME) (https://vizhub.healthdata.org/gbd-results/). GBD 2023 quantifies health loss attributable to diseases and injuries across countries and over time. GBD 2023 provides estimates for 375 diseases and injuries and 88 risk factors by age and sex across 204 countries and territories, including 660 subnational locations (14, 15). Within the GBD cause hierarchy, transport injuries (TIs) are Level 2 conditions under injuries. TIs comprise road injuries—subdivided into motor-vehicle, cyclist, motorcyclist, pedestrian and other road injuries—and other transport injuries.

We extracted incidence, prevalence, deaths and DALYs attributable to TIs for people aged <20 years across the 33 WPR countries and territories. Rates were expressed per 100,000 population. Temporal trends from 2000 to 2023 were assessed using annual percentage change (APC) with 95% confidence intervals (CIs). Reporting followed the Guidelines for Accurate and Transparent Health Estimates Reporting (GATHER) (16). Indonesia was formally transferred from the World Health Organization (WHO) South-East Asia Region to the WPR in 2025 (17); it was therefore included throughout the analysis. All analyses were conducted using R version 4.5.2 (R Core Team, 2025; R Foundation for Statistical Computing, Vienna, Austria).

### Temporal trend analysis

Temporal trends in TI burden from 2000 to 2023 were assessed across four indicators—incidence, prevalence, mortality, and DALYs—among children and adolescents aged <20 years in the WPR. For each indicator, absolute numbers and age-standardised rates (ASRs) per 100,000 population were plotted as dual-axis charts, with counts displayed as bar charts on the primary axis and rates as line graphs on the secondary axis. ASRs were calculated by direct standardisation against the Global Burden of Disease reference population to remove the confounding effect of age-structure differences (14, 15). Details of the formula and weights are provided in the Supplementary Material S1 (18).

### Statistical analyses

For 2023, we compared the age-standardised incidence rate (ASIR), age-standardised prevalence rate (ASPR) and age-standardised mortality rate (ASMR) across the 33 locations. Geographic distributions were displayed using six colour classes. Absolute numbers and corresponding age-standardised rates for incidence, prevalence and mortality were tabulated by location.

TI burden estimates were stratified by four age groups (<5, 5–9, 10–14, and 15–19 years) and by sex. For each stratum, we reported absolute numbers and age-specific rates per 100,000 population, with 95% uncertainty intervals (UIs), for incidence, prevalence, mortality, and DALYs. Sex- and age-specific disparities were visualized using grouped bar charts for absolute numbers and overlaid line graphs for age-specific rates.

Changes in TI burden from 2000 to 2023 were decomposed into three additive components: population growth, population aging, and epidemiological change. The decomposition was applied to absolute numbers of incident cases, prevalent cases, deaths, and DALYs, and was performed for both sexes combined and separately for males and females. Each component quantifies the net change attributable to the corresponding factor, holding the other two constant. We computed the three components from age-specific population shares aᵢₜ, total population counts pₜ, and age-specific DALY rates rᵢₜ (per 10^5^ persons) using the exact algebraic formulation now provided in Supplementary Methods S2. The overall change in DALYs equals the sum of the three effects, and percentage contributions were derived accordingly (14, 19).

Future trends in age-standardised TI burden rates from 2024 to 2035 were projected using sex-specific autoregressive integrated moving average (ARIMA) models. ARIMA integrates autoregressive and moving-average components, accommodates autocorrelation in time-series analysis data, and can be selected using information criteria (20, 21). Model orders (p, d, q) were identified by minimizing the corrected Akaike information criterion (AICc), and the adequacy of the selected models was confirmed using Ljung–Box tests of residual autocorrelation. Stationarity of each series was assessed with the augmented Dickey–Fuller (ADF) test, with differencing applied as necessary to achieve stationarity. Projections were performed separately by sex for all four burden indicators—incidence, prevalence, mortality, and DALYs—with 95% confidence intervals, using historical trends from 2000 to 2023 as the basis for model fitting. Because linear ARIMA forecasts are not constrained to non-negative values and may extrapolate across zero under strong downward trends, the projection horizon was restricted to 2024–2035, the period over which the forecasts and their 95% confidence intervals remained strictly positive and epidemiologically plausible; full modeling details are provided in the Supplementary Methods S3. Sensitivity analyses using log-transformed ARIMA models were additionally performed to assess robustness.

## Results

### Temporal trends in TI burden, 2000–2023

Transport-injury incidence among people aged <20 years in the WPR was 5,034,407 cases (95% UI, 4,180,502–5,955,077) in 2000. It fell to 2,433,402 (1,988,777–2,920,981) in 2023, a 51.7% reduction. The ASIR decreased by 47.2%, from 812.69 (605.12–1,065.33) to 428.74 (315.68–560.86) per 100,000. The corresponding APC was −2.82% (95% CI, −3.04 to −2.61; *P* < 0.001) (Table 1). All 33 locations had negative APC estimates. The decline was steepest in the Republic of Korea (APC, −4.72%; 95% CI, −5.20 to −4.23). It was least steep in the Northern Mariana Islands (−0.22%; −0.29 to −0.15). In 2023, the regional burden still included approximately 1,468,431 prevalent cases, 28,379 deaths, and 2,282,094 DALYs. Prevalence, mortality, and DALY rates also declined throughout the study period (Figure 1).

**Table 1:** Incidence rates, age-standardized rates, and annual percentage changes in transport injuries among the Western Pacific Region population aged <20 years, from 2000 to 2023.

Location	2000		2023		APC of ASR (95% CI)	*P* value
	No. incident cases (95% UI)	ASIR per 100,000 (95% UI)	No. incident cases (95%UI)	ASIR per 100,000 (95%UI)		
Western Pacific Region	5,034,407 (4,180,502, 5,955,077)	812.69 (605.12, 1,065.33)	2,433,402 (1,988,777, 2,920,981)	428.74 (315.68, 560.86)	−2.82 (−3.04, −2.61)	<0.001
China	3,221,400 (2,600,337, 3,901,493)	837.94 (602.87, 1,130.8)	1,504,110 (1,194,163, 1,839,537)	452.29 (321.52, 606.06)	−2.62 (−2.81, −2.42)	<0.001
Taiwan (Province of China)	50,778 (46,949, 55,365)	704.66 (621.99, 798.96)	17,539 (15,855, 19,959)	431.56 (356.56, 518.57)	−2.45 (−2.74, −2.15)	<0.001
Japan	253,601 (202,930, 311,849)	916.24 (649.74, 1,233.36)	105,198 (85,879, 129,396)	504.69 (357.04, 685.18)	−2.95 (−3.29, −2.61)	<0.001
Mongolia	6,906 (6,119, 7,730)	617.51 (505.85, 730.71)	6,308 (5,678, 7,027)	465.9 (378.94, 556.94)	−1.33 (−1.64, −1.02)	<0.001
Republic of Korea	263,414 (244,013, 288,202)	1,862.07 (1,629.91, 2,128.45)	56,093 (48,671, 65,185)	716.04 (567.83, 879.27)	−4.72 (−5.20, −4.23)	<0.001
Brunei Darussalam	2,001 (1,818, 2,192)	1,462.17 (1,248.92, 1,722.94)	1,252 (1,116, 1,389)	971.55 (795.61, 1,167.6)	−2.06 (−2.40, −1.72)	<0.001
Cambodia	36,774 (32,952, 40,662)	562 (466.87, 672.16)	23,207 (21,102, 25,550)	350.95 (292.94, 413.55)	−2.24 (−2.41, −2.07)	<0.001
Lao People's Democratic Republic	17,951 (16,283, 20,264)	622.66 (514, 750.37)	10,295 (9,257, 11,405)	345.42 (286.05, 409.91)	−2.89 (−3.06, −2.71)	<0.001
Malaysia	52,611 (46,994, 58,240)	494.43 (419.12, 580.57)	44,370 (39,333, 49,650)	395.42 (324.23, 476.79)	−0.99 (−1.10, −0.88)	<0.001
Philippines	124,902 (100,356, 148,199)	325.83 (231.05, 441.18)	111,248 (89,774, 134,552)	248.37 (175.07, 332.71)	−1.38 (−1.50, −1.26)	<0.001
Singapore	8,757 (7,640, 9,956)	812.16 (641.72, 1,002.73)	6,166 (5,003, 7,393)	616.05 (454.95, 813.93)	−1.37 (−1.60, −1.14)	<0.001
Viet Nam	159,057 (143,544, 179,229)	447.81 (373.36, 534.48)	129,628 (116,619, 142,939)	406.2 (340.59, 476.75)	−0.71 (−1.12, −0.31)	0.001
Indonesia	789,748 (677,193, 919,556)	901.42 (670.05, 1,174.43)	356,183 (294,357, 429,275)	387.66 (280.28, 513.01)	−3.97 (−4.34, −3.59)	<0.001
Australia	56,061 (51,174, 61,848)	1,012.29 (848.18, 1,195.44)	39,066 (34,328, 44,804)	588.7 (465.22, 728.81)	−2.76 (−2.96, −2.55)	<0.001
New Zealand	20,750 (16,908, 25,188)	1,761.23 (1,284.67, 2,324.45)	10,796 (8,770, 13,156)	793.4 (570.86, 1,061.85)	−4.00 (−4.83, −3.16)	<0.001
Fiji	1,189 (1,060, 1,331)	333.19 (271.4, 397.86)	986 (874, 1,096)	278.65 (225.74, 336.38)	−0.97 (−1.16, −0.79)	<0.001
Papua New Guinea	17,080 (15,233, 18,847)	592.25 (486.5, 708.35)	25,974 (23,516, 28,556)	492.72 (407.75, 586.9)	−0.94 (−1.01, −0.87)	<0.001
Solomon Islands	713 (629, 820)	309.59 (242.29, 390.27)	1,157 (1,008, 1,332)	285.75 (226.4, 356.76)	−0.40 (−0.48, −0.33)	<0.001
Vanuatu	426 (378, 472)	420.53 (340.05, 515.24)	549 (493, 607)	365.94 (296.51, 437.14)	−0.80 (−0.89, −0.71)	<0.001
Guam	224 (201, 248)	364.12 (301.12, 433.38)	136 (120, 153)	285.97 (231, 345.65)	−1.22 (−1.39, −1.05)	<0.001
Kiribati	148 (131, 166)	340.12 (273.06, 416.54)	151 (133, 169)	275.1 (221.88, 335.33)	−1.26 (−1.45, −1.07)	<0.001
Marshall Islands	108 (96, 120)	368.99 (296.8, 444.94)	54 (47, 60)	289.93 (233.32, 351.84)	−1.36 (−1.52, −1.20)	<0.001
Micronesia (Federated States of)	245 (218, 276)	427.01 (348.46, 517.44)	139 (123, 156)	327.82 (263.24, 405.16)	−1.23 (−1.35, −1.11)	<0.001
Nauru	34 (30, 37)	640.88 (522.71, 769.05)	29 (26, 32)	502.95 (420.31, 593.36)	−1.49 (−1.69, −1.29)	<0.001
Northern Mariana Islands	73 (65, 82)	338.47 (265.7, 413.72)	44 (39, 50)	313.05 (252.41, 379.91)	−0.22 (−0.29, −0.15)	<0.001
Palau	25 (22, 28)	400.36 (317.86, 488.75)	15 (13, 17)	338.16 (272.48, 410.72)	−0.79 (−0.86, −0.73)	<0.001
American Samoa	74 (66, 84)	270.9 (213.41, 331.93)	41 (36, 47)	219.59 (170.17, 272.81)	−1.13 (−1.27, −0.99)	<0.001
Cook Islands	34 (31, 38)	493.28 (416.9, 582.65)	13 (12, 15)	262.48 (205.17, 330.21)	−3.32 (−3.58, −3.05)	<0.001
Niue	3 (3, 3)	410.32 (329.39, 497.46)	2 (2, 2)	344.08 (279.92, 415.98)	−0.93 (−1.03, −0.83)	<0.001
Samoa	307 (271, 347)	345.27 (276.24, 423.71)	286 (252, 326)	283.65 (225.77, 347.37)	−0.91 (−1.00, −0.83)	<0.001
Tokelau	2 (2, 2)	311.96 (244.51, 386.83)	1 (1, 1)	216.97 (165.21, 276.13)	−1.92 (−2.09, −1.74)	<0.001
Tonga	143 (126, 159)	279.67 (225.98, 338.8)	112 (99, 127)	236.12 (186.93, 290.25)	−0.96 (−1.06, −0.86)	<0.001
Tuvalu	20 (17, 22)	475.96 (389.26, 577.21)	13 (12, 15)	301.99 (242.05, 369.01)	−2.15 (−2.29, −2.01)	<0.001

ASIR, age-standardized incidence rate; APC, annual percent change; CI, confidence interval; UI, uncertainty interval.

**Figure 1:**
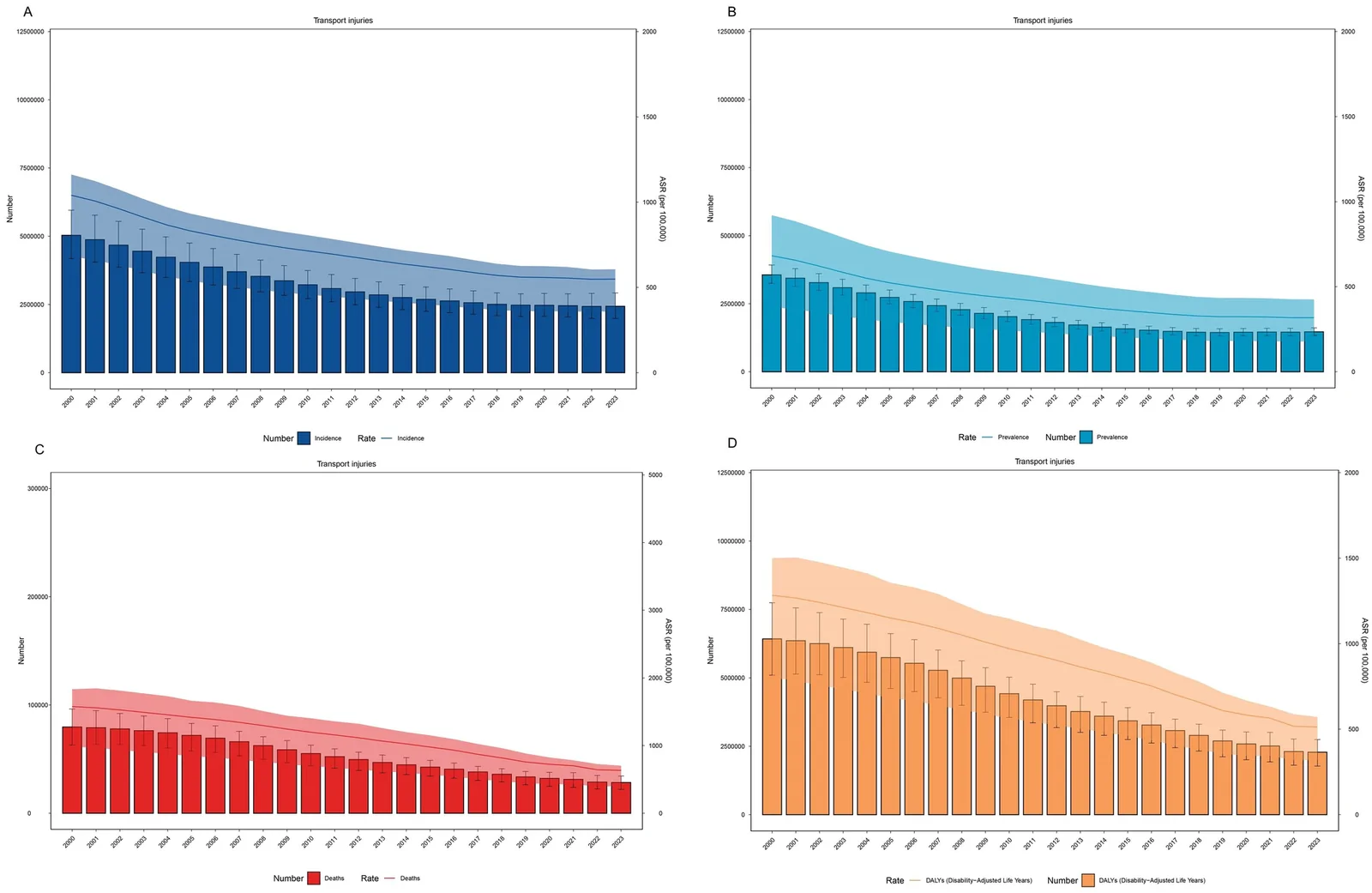
Temporal trends in transport injury burden among individuals aged <20 years in the Western Pacific Region, 2000–2023. Panels display **(A)** incidence, **(B)** prevalence, **(C)** deaths, and **(D)** DALYs. Bars represent absolute numbers (left axis); lines represent age-standardized rates per 100,000 population (right axis). DALYs, disability-adjusted life years.

### Geographic heterogeneity in TI burden, 2023

Marked geographic variation in TI burden was observed across the WPR in 2023 (Figure 2, Supplementary Table A). ASIR ranged from 216.97 per 100,000 in Tokelau to 971.55 per 100,000 in Brunei Darussalam, a 4.48-fold difference. High-income settings exhibited divergent patterns: the Republic of Korea and Brunei Darussalam recorded the highest ASIR (716.04 and 971.55, respectively), whereas Singapore and Japan had intermediate rates (616.05 and 504.69). Pacific island settings also showed marked variation, with ASIRs of 502.95 per 100,000 in Nauru and 219.59 per 100,000 in American Samoa. ASMR varied 38-fold, from 0.61 per 100,000 (Singapore) to 23.28 per 100,000 (Nauru). Indonesia and Cambodia bore the highest ASMR among larger populations (10.72 and 10.56, respectively, vs. the WPR average of 4.94). In absolute terms, China accounted for 61.8% of all incident cases (1,504,110 of 2,433,402) and 39.0% of deaths (11,073 of 28,379), reflecting its population size despite moderate age-standardized rates.

**Figure 2:**
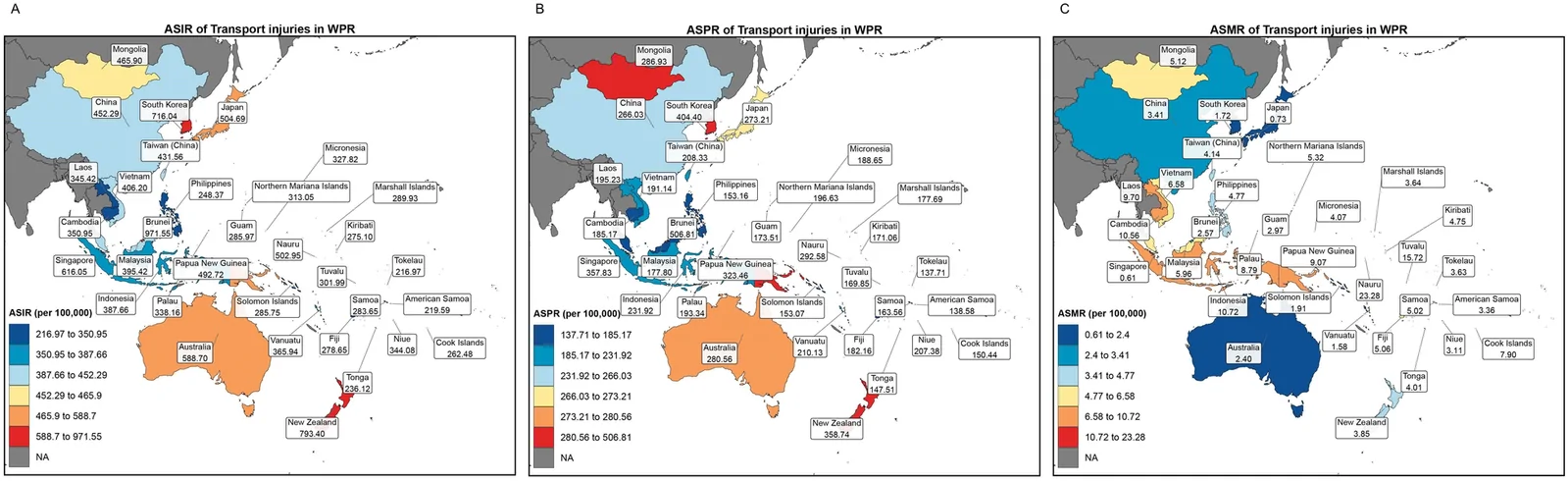
Geographic distribution of transport injury burden among individuals aged <20 years across the Western Pacific Region in 2023. Maps display age-standardized rates per 100,000 population for **(A)** incidence (ASIR), **(B)** prevalence (ASPR), and **(C)** death (ASMR), using six colour classes to indicate rate ranges. ASIR, age-standardized incidence rate; ASPR, age-standardized prevalence rate; ASMR, age-standardized mortality rate.

### Age and sex disparities in TI burden

Rates increased across the four age groups and were highest at ages 15–19 years (Figure 3; Supplementary Tables B1–B4). In that age group, the incidence rate was 996.81 per 100,000 (95% UI, 718.74–1,340.36) in males and 434.88 (309.46–578.32) in females. Mortality rates were 14.27 and 5.66 per 100,000, respectively, and DALY rates were 1,068.25 and 432.21 per 100,000. At ages 0–4 years, incidence rates were similar in females and males (402.33 and 383.29 per 100,000). Sex differences widened across later age groups, particularly for mortality and DALYs.

**Figure 3:**
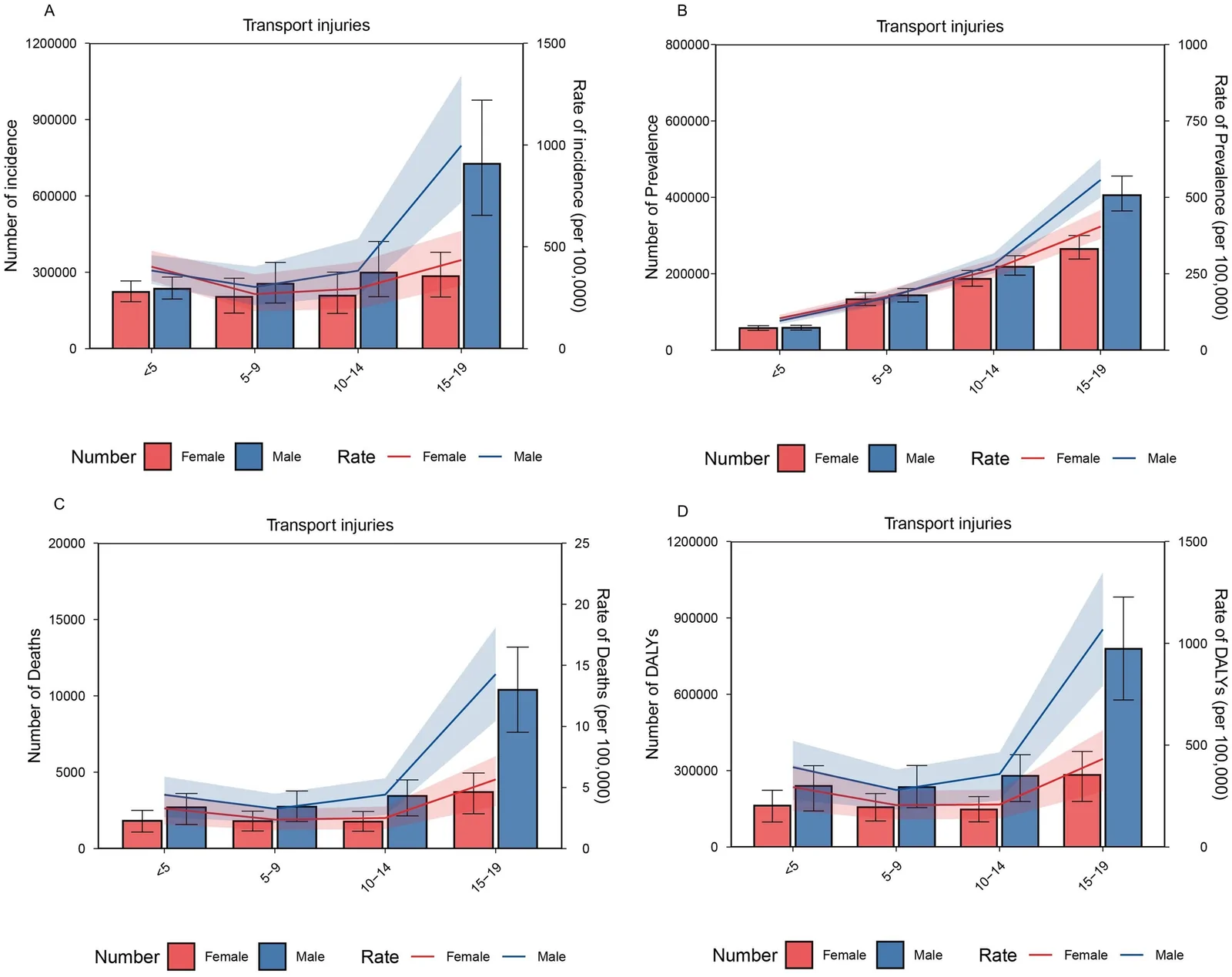
Age- and sex-specific burden of transport injuries among individuals aged <20 years in the Western Pacific Region. Panels display **(A)** incidence, **(B)** prevalence, **(C)** deaths, and **(D)** DALYs, stratified by age group (<5, 5–9, 10–14, and 15–19 years) and sex. Bars represent absolute numbers; lines represent age-specific rates per 100,000 population. DALYs, disability-adjusted life years.

### Drivers of TI burden change: decomposition analysis

Decomposition of the 2000–2023 changes in TI burden revealed distinct contributions of population growth, population aging, and epidemiological change across the four indicators (Figure 4 and Supplementary Material-Decomposition Analysis.csv). For incidence and deaths, epidemiological change—reflecting declines in age- and sex-specific rates—was the predominant driver of reduction, while population growth partially offset these declines, particularly among males. Population aging contributed a comparatively modest decrement. For prevalence and DALYs, the net reduction was more attenuated, as population growth exerted a stronger countervailing effect. Across all indicators, the absolute contribution of epidemiological improvement was greater in males than in females, consistent with the steeper rate declines observed in male adolescents.

**Figure 4:**
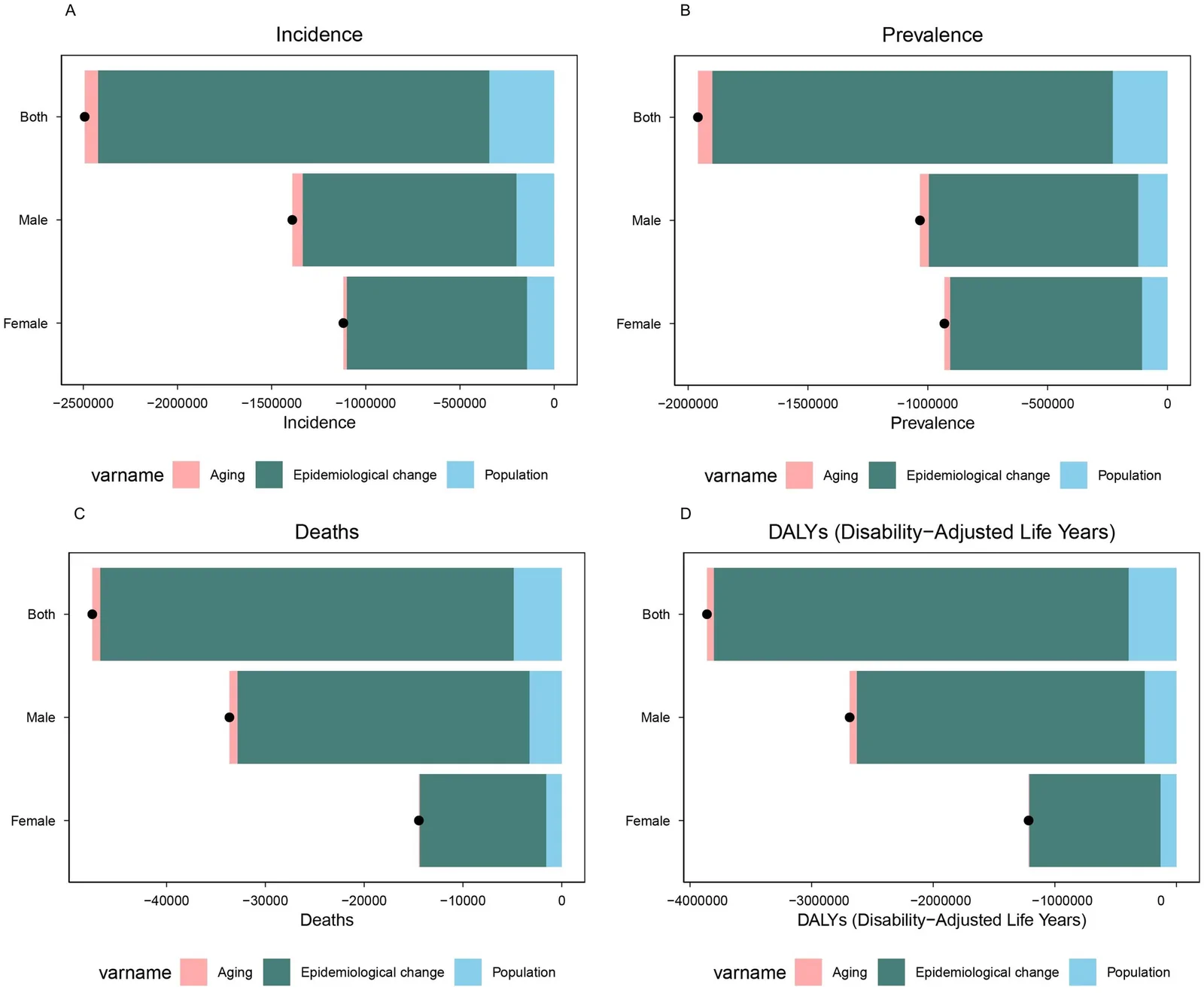
Decomposition of changes in transport injury burden among individuals aged <20 years in the Western Pacific Region from 2000 to 2023. Panels show the decomposition for **(A)** incidence, **(B)** prevalence, **(C)** deaths, and **(D)** DALYs. DALYs, disability-adjusted life years.

### Projected TI burden, 2024–2035

ARIMA projections indicated continued declines in TI burden through 2035, with marked sex-specific differences (Figure 5, Supplementary Tables C1, C2). Among males, all four indicators declined between 2024 and 2035: the incidence rate fell from 497.96 to 466.24 per 100,000, the prevalence rate from 262.45 to 240.29 per 100,000, and the mortality rate from 5.89 to 1.17 per 100,000, while the DALY rate declined substantially from 471.41 to 93.39 per 100,000. Among females, incidence and prevalence remained essentially stable (from 349.33 to 350.83 and from 229.85 to 227.02 per 100,000, respectively), whereas mortality and DALY rates declined progressively, from 3.13 to 0.83 and from 258.11 to 62.50 per 100,000, respectively. Overall, the sex gap in TI-related mortality and DALYs was projected to narrow, although male DALYs remained substantially elevated relative to females through the end of the projection period. Sensitivity analyses using log-transformed ARIMA (1,1,1) models yielded incidence and prevalence trajectories consistent with the primary models (within ±14% at 2035), but more conservative mortality and DALY projections (143%–279% higher at 2035; Supplementary Tables C3, C4, Supplementary Figure S1). These results indicate that the near-zero mortality and DALY projections are sensitive to the assumption of an additive linear decline.

**Figure 5:**
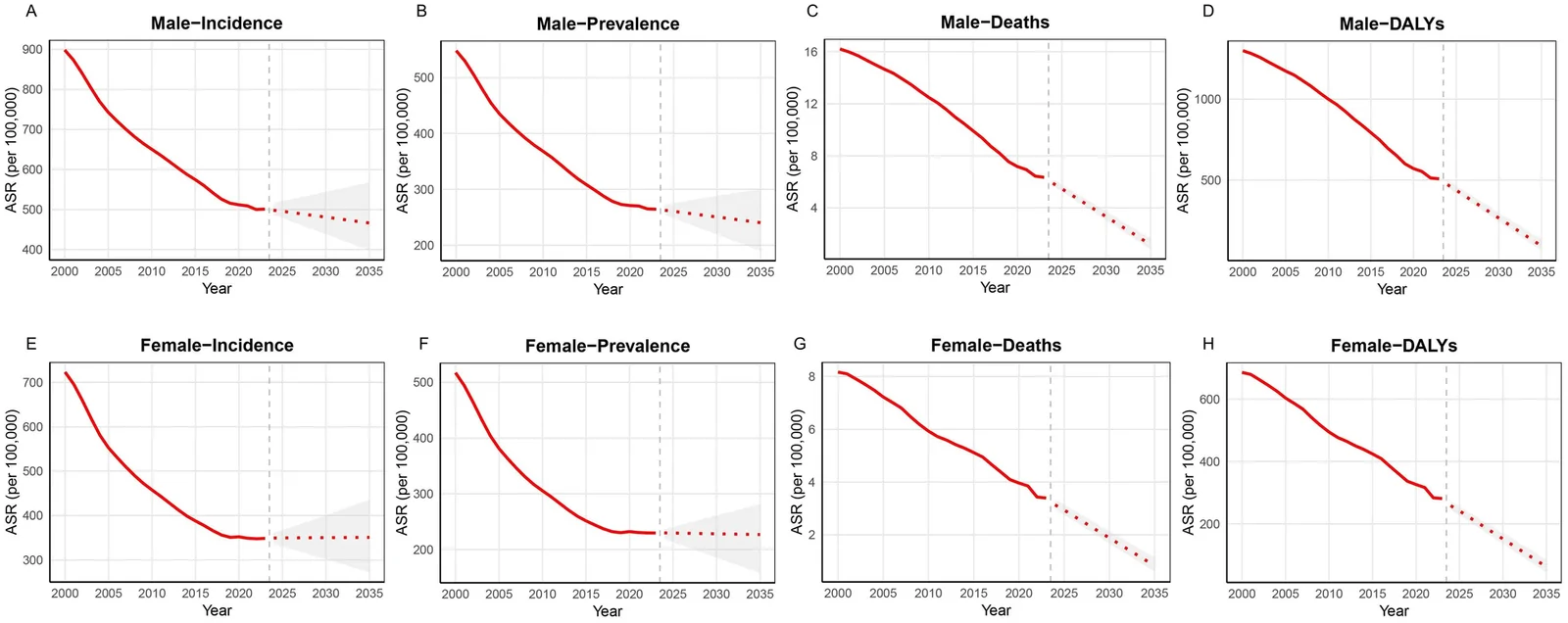
Age-standardized rates of transport injury burden among individuals aged <20 years in the Western Pacific Region, 2000–2035. Panels **A–D** display projections for males: **(A)** incidence, **(B)** prevalence, **(C)** deaths, and **(D)** DALYs. Panels **E–H** display projections for females: **(E)** incidence, **(F)** prevalence, **(G)** deaths, and **(H)** DALYs. Solid lines represent observed rates (2000–2023); dashed lines represent ARIMA-model projections (2024–2035) with 95% confidence intervals (shaded areas). ASR, age-standardized rate; DALYs, disability-adjusted life years.

## Discussion

Transport-injury burden in the WPR declined between 2000 and 2023, but the remaining caseload was large. Both incident cases and age-standardised incidence rates fell, yet an estimated 2.43 million cases occurred in 2023. Mortality rates varied far more across locations than incidence rates, and rankings by rate did not match rankings by absolute number. Males aged 15–19 years had the highest incidence, mortality and DALY rates.

The downward regional trend resembles previous reports of declining age-standardised road-injury mortality and DALY rates (1, 4–7, 9). Changes in travel exposure, road-user mix, road and vehicle safety, enforcement, and trauma care may all have contributed. Their individual contributions cannot be estimated because the data lack location-specific measures of exposure, policy implementation and care pathways.

Projected trends differed between fatal and non-fatal outcomes. Incidence and prevalence declined modestly in males but remained broadly stable in females, whereas mortality and DALY rates fell in both sexes. Comparable ARIMA approaches have been used to project GBD-based estimates of dementia in Japan and cardiometabolic liver disease in Asia (20, 21). These forecasts suggest that deaths may continue to decline without a comparable reduction in non-fatal injury. Nevertheless, log-transformed ARIMA sensitivity analyses suggested that the projected near-elimination of mortality and DALYs should be interpreted with caution. Prevention and rehabilitation services will therefore remain necessary, particularly for older adolescent males, while stable incidence and prevalence among females merit continued monitoring.

Geographic differences in mortality may reflect variation in road-user mix, speed environments, helmet and restraint use, injury severity, emergency-care access and death registration (22). Prehospital trauma care remains limited in many low- and middle-income settings (23). Evidence on the effectiveness of organised trauma systems is less consistent and appears to depend on context (24). Rankings for small territories require particular caution because a small number of events can produce high rates and wide UIs. Rates describe burden relative to population size, whereas counts more directly indicate the likely scale of prevention and service needs.

The age and sex profiles were also distinct. The higher burden in males is consistent with broader evidence on sex differences in health loss and crash outcomes (25, 26). Incidence rates were similar between males and females at ages 0–4 years but diverged across later age groups. Greater mobility, road exposure and risk-taking during adolescence could contribute to this difference. Studies in Chinese adolescents have associated traffic injury with road-safety attitudes, behaviour, exposure and sensation seeking (27, 28). Alcohol is also an established contributor to injury burden worldwide (29). The GBD estimates contain no behavioural or exposure data with which to examine these explanations across the WPR.

The diversity of transport environments across the WPR argues for locally selected measures rather than a uniform regional programme. Available evidence supports packages combining legislation and enforcement with safer road design, education and post-crash care (30–34). The appropriate mix depends on local road-user and injury-mechanism data. Relevant measures include motorcycle helmet use, occupant restraints, pedestrian protection, graduated licensing and alcohol control (35–41). Experience from China and Cambodia indicates that legislation has limited value without sustained implementation and enforcement (42, 43).

The regional patterns also resemble those reported in recent global analyses. An all-injury study found a greater burden in males, peak incidence at ages 15–19 years and road traffic injury among the leading causes in people aged <20 years (44). The GBD 2023 road-injury analysis reported declining global incidence and mortality, although progress differed across income groups (45). The category examined here includes both road injuries and other transport injuries. These aggregate estimates cannot, by themselves, determine which interventions are most appropriate for an individual location.

Analysing incidence, prevalence, mortality and DALYs within the same GBD framework allowed fatal and non-fatal outcomes to be compared on a common regional scale. Comparing rates with counts showed that relative burden and service caseload do not necessarily identify the same locations. The age- and sex-specific analyses also identified males aged 15–19 years as the group with the highest rates across several outcomes.

Interpretation depends on the quality and structure of the underlying GBD inputs. The estimates draw on surveillance, census and vital-registration data that differ in completeness across locations. Uncertainty is greater where observations are sparse, and single-year rankings may overstate differences among small territories. The regional series also applies current WHO membership retrospectively. Comparisons between regional totals and location-level sums require consistent treatment of the GBD hierarchy, particularly for China and Taiwan.

The aggregate transport-injury category provides no information on road-user type or injury mechanism. The extracted data also contain no direct measures of travel exposure, policy implementation, prehospital care or in-hospital treatment. A single APC for 2000–2023 may conceal non-linear or recent changes, and uncertainty in the GBD estimates was not propagated through the APC calculation. Further work will require road-user-specific surveillance linked to exposure and care-pathway data.

## Conclusion

Transport-injury burden among children and adolescents in the WPR declined markedly between 2000 and 2023, largely because age- and sex-specific rates fell. However, progress remained uneven across locations, and males aged 15–19 years retained the greatest burden. The 2024–2035 ARIMA projections suggest modest declines in male age-standardised incidence and prevalence rates, whereas corresponding female rates may remain broadly stable. These trends imply that past regional gains may not eliminate persistent inequalities without geographically targeted road-safety systems and age-appropriate measures for older adolescent males. The projections offer a directional signal for surveillance and planning, while future model refinement should strengthen their use in policy evaluation.

## Data Availability

The data analysed in this study are publicly available through the Global Health Data Exchange and the GBD Results Tool (https://vizhub.healthdata.org/gbd-results/). Derived results supporting the conclusions are provided in the Supplementary Tables. Further inquiries may be directed to the corresponding authors.

## References

[1] Peden AE Cullen P Francis KL Moeller H Peden MM Ye P. Adolescent transport and unintentional injuries: a systematic analysis using the global burden of disease study 2019. Lancet Public Health. (2022) 7:e657–69. 10.1016/S2468-2667(22)00134-7 35779567 PMC9329128

[2] Ward JL Azzopardi PS Francis KL Santelli JS Skirbekk V Sawyer SM. Global, regional, and national mortality among young people aged 10–24 years, 1950–2019: a systematic analysis for the global burden of disease study 2019. Lancet. (2021) 398:1593–618. 10.1016/S0140-6736(21)01546-4 34755628 PMC8576274

[3] Liu L Villavicencio F Yeung D Perin J Lopez G Strong KL. National, regional, and global causes of mortality in 5–19-year-olds from 2000 to 2019: a systematic analysis. Lancet Glob Health. (2022) 10:e337–47. 10.1016/S2214-109X(21)00566-0 35180417 PMC8864304

[4] Khan UR Razzak JA Wärnberg MG. Global trends in adolescents’ road traffic injury mortality, 1990–2019. Arch Dis Child. (2021) 106:753–7. 10.1136/archdischild-2020-319184 33597185 PMC8311071

[5] James SL Lucchesi LR Bisignano C Castle CD Dingels ZV Fox JT. Morbidity and mortality from road injuries: results from the global burden of disease study 2017. Inj Prev. (2020) 26:i46–56. 10.1136/injuryprev-2019-043302 31915274 PMC7571357

[6] Xu Y Chen M Yang R Wumaierjiang M Huang S. Global, regional, and national burden of road injuries from 1990 to 2019. Int J Environ Res Public Health. (2022) 19:16479. 10.3390/ijerph192416479 36554366 PMC9779128

[7] Wan R Xia J Duan F Min L Liu T. Global burden and trends of transport injuries from 1990 to 2019: an observational trend study. Inj Prev. (2023) 29:418–24. 10.1136/ip-2023-044915 37549986 PMC10579470

[8] Du W Fan H Li H Hao C. Trends in road traffic injury mortality and age-period-cohort analysis among children and adolescents in China and globally. Traffic Inj Prev. (2026) 27:462–71. 10.1080/15389588.2025.2528147 40680209

[9] Song Z Zhang B Pang S Qiu M Huang J Hao J. Trends in the burden of road traffic injuries among children and adolescents aged 0–19 years in low- and middle-income countries, 1990–2023. J Glob Health. (2026) 16:04094. 10.7189/jogh.16.04094 41818628 PMC12981736

[10] Herman J Ameratunga S Jackson R. Burden of road traffic injuries and related risk factors in low and middle-income Pacific island countries and territories: a systematic review of the scientific literature (TRIP 5). BMC Public Health. (2012) 12:479. 10.1186/1471-2458-12-479 22731963 PMC3490885

[11] Abdul-Razak S Azzopardi PS Patton GC Mokdad AH Sawyer SM. Child and adolescent mortality across Malaysia’s epidemiological transition: a systematic analysis of global burden of disease data. J Adolesc Health. (2017) 61:424–33. 10.1016/j.jadohealth.2017.05.014 28838752

[12] Liu Y Chu Y Yeung D Wang W Wang L Yin P. National and sub-national levels and causes of mortality among 5–19-year-olds in China in 2004–2019: a systematic analysis of evidence from the disease surveillance points system. J Glob Health. (2022) 12:11008. 10.7189/jogh.12.11008 36181508 PMC9526435

[13] Hyder AA Muzaffar SSF Bachani AM. Road traffic injuries in urban Africa and Asia: a policy gap in child and adolescent health. Public Health. (2008) 122:1104–10. 10.1016/j.puhe.2007.12.014 18597800

[14] Hay SI Ong KL Santomauro DF Bhoomadevi A Aalipour MA Aalruz H. Burden of 375 diseases and injuries, risk-attributable burden of 88 risk factors, and healthy life expectancy in 204 countries and territories, including 660 subnational locations, 1990–2023: a systematic analysis for the global burden of disease study 2023. Lancet. (2025) 406:1873–922. 10.1016/S0140-6736(25)01637-X 41092926 PMC12535840

[15] Naghavi M Kyu HH Bhoomadevi B Aalipour MA Aalruz H Ababneh HS. Global burden of 292 causes of death in 204 countries and territories and 660 subnational locations, 1990–2023: a systematic analysis for the global burden of disease study 2023. Lancet. (2025) 406:1811–72. 10.1016/S0140-6736(25)01917-8 41092928 PMC12535838

[16] Stevens GA Alkema L Black RE Boerma JT Collins GS Ezzati M. Guidelines for accurate and transparent health estimates reporting: the GATHER statement. Lancet. (2016) 388:e19–23. 10.1016/S0140-6736(16)30388-9 27371184

[17] World Health Organization. Reassignment of Indonesia from the south-east Asia region to the Western Pacific Region. Resolution WHA78.25. Geneva: World Health Organization (2025). Available online at: https://apps.who.int/gb/ebwha/pdf_files/WHA78/A78_R25-en.pdf (Accessed August 29, 2026).

[18] Yang K Yang X Jin C Ding S Liu T Ma B. Global burden of type 1 diabetes in adults aged 65 years and older, 1990–2019: population based study. Br Med J. (2024) 385:e078432. 10.1136/bmj-2023-078432 PMC11167563 38866425

[19] Das Gupta P. Standardization and Decomposition of Rates: A User’s Manual. Current Population Reports, Series P23-186. Washington, DC: U.S. Bureau of the Census, U.S. Government Printing Office (1993).

[20] Behera DK Rahut DB Tripathy S. Alzheimer’s disease and dementia in Japan: epidemiological trends, regional disparities, and future projections. Alzheimers Dement. (2025) 21:e70444. 10.1002/alz.70444 40631426 PMC12238829

[21] Duo T Wen Y Bian Y Wang Y Zhang X Ju J. Rising burden of MASLD and CKM syndrome in Asia: a decade of trends and future projections. Metab Clin Exp. (2026) 178:156549. 10.1016/j.metabol.2026.156549 41643809

[22] Giummarra MJ Lau G Grant G Gabbe BJ. A systematic review of the association between fault or blame-related attributions and procedures after transport injury and health and work-related outcomes. Accid Anal Prev. (2020) 135:105333. 10.1016/j.aap.2019.105333 31863937

[23] Quake SYL Khoda F Arjomandi Rad A Subbiah Ponniah H Vardanyan R Frisoni P. The current status and challenges of prehospital trauma care in low- and middle-income countries: a systematic review. Prehosp Emerg Care. (2024) 28:76–86. 10.1080/10903127.2023.2165744 36629481

[24] Mwandri M Stewart B Hardcastle TC Hudson J Rubiano AM Gruen RL. Organised trauma systems and designated trauma centres for improving outcomes in injured patients. Cochrane Database Syst Rev. (2025) 8:CD012500. 10.1002/14651858.CD012500.pub2 40747779 PMC12315082

[25] Patwardhan V Gil GF Arrieta A Cagney J DeGraw E Herbert ME. Differences across the lifespan between females and males in the top 20 causes of disease burden globally: a systematic analysis of the global burden of disease study 2021. Lancet Public Health. (2024) 9:e282–94. 10.1016/S2468-2667(24)00053-7 38702093 PMC11080072

[26] Craig MJ Liu C Zhang F Enriquez J. Sex-based differences in odds of motor vehicle crash injury outcomes. Accid Anal Prev. (2024) 195:107100. 10.1016/j.aap.2023.107100 38154856

[27] Wang H Wu M Cheng X Schwebel DC. The road user behaviours of Chinese adolescents: data from China and a comparison with adolescents in other countries. Ann Glob Health. (2019) 85:76. 10.5334/aogh.2452 31148438 PMC6634457

[28] Wang H Shi L Schwebel DC. Relations between adolescent sensation seeking and traffic injury: multiple-mediating effects of road safety attitudes, intentions and behaviors. Traffic Inj Prev. (2019) 20:789–95. 10.1080/15389588.2019.1666982 31738575 PMC10413049

[29] Griswold MG Fullman N Hawley C Arian N Zimsen SRM Tymeson HD. Alcohol use and burden for 195 countries and territories, 1990–2016: a systematic analysis for the global burden of disease study 2016. Lancet. (2018) 392:1015–35. 10.1016/S0140-6736(18)31310-2 30146330 PMC6148333

[30] Peden AE Cullen P Bhandari B Testa L Wang A Ma T. A systematic review of the evidence for effectiveness of interventions to address transport and other unintentional injuries among adolescents. J Safety Res. (2023) 85:321–38. 10.1016/j.jsr.2023.03.005 37330882

[31] Salam RA Arshad A Das JK Khan MN Mahmood W Freedman SB. Interventions to prevent unintentional injuries among adolescents: a systematic review and meta-analysis. J Adolesc Health. (2016) 59:S76–87. 10.1016/j.jadohealth.2016.07.024 27664598 PMC5026686

[32] Fisa R Musukuma M Sampa M Musonda P Young T. Effects of interventions for preventing road traffic crashes: an overview of systematic reviews. BMC Public Health. (2022) 22:513. 10.1186/s12889-021-12253-y 35296294 PMC8925136

[33] Staton C Vissoci J Gong E Toomey N Wafula R Abdelgadir J. Road traffic injury prevention initiatives: a systematic review and metasummary of effectiveness in low and middle income countries. PLoS One. (2016) 11:e0144971. 10.1371/journal.pone.0144971 26735918 PMC4703343

[34] Akbari M Heydari ST Razzaghi A Vali M Tabrizi R Bagheri Lankarani K. Effectiveness of interventions for preventing road traffic injuries: a systematic review in low-, middle- and high-income countries. PLoS One. (2024) 19:e0312428. 10.1371/journal.pone.0312428 39636886 PMC11620428

[35] Lepard JR Spagiari R Corley J Barthélemy EJ Kim E Patterson R. Differences in outcomes of mandatory motorcycle helmet legislation by country income level: a systematic review and meta-analysis. PLoS Med. (2021) 18:e1003795. 10.1371/journal.pmed.1003795 34534215 PMC8486090

[36] Liu BC Ivers R Norton R Boufous S Blows S Lo SK. Helmets for preventing injury in motorcycle riders. Cochrane Database Syst Rev. (2008) 1:CD004333. 10.1002/14651858.CD004333.pub3 PMC13092890 18254047

[37] Fouda Mbarga N Abubakari AR Aminde LN Morgan AR. Seatbelt use and risk of major injuries sustained by vehicle occupants during motor-vehicle crashes: a systematic review and meta-analysis of cohort studies. BMC Public Health. (2018) 18:1413. 10.1186/s12889-018-6280-1 30594164 PMC6310927

[38] Namatovu S Balugaba BE Muni K Ningwa A Nsabagwa L Oporia F. Interventions to reduce pedestrian road traffic injuries: a systematic review of randomized controlled trials, cluster randomized controlled trials, interrupted time-series, and controlled before-after studies. PLoS One. (2022) 17:e0262681. 10.1371/journal.pone.0262681 35073351 PMC8786203

[39] Zhu M Cummings P Chu H Coben JH Li G. Graduated driver licensing and motor vehicle crashes involving teenage drivers: an exploratory age-stratified meta-analysis. Inj Prev. (2013) 19:49–57. 10.1136/injuryprev-2012-040474 23211352 PMC4103686

[40] Russell KF Vandermeer B Hartling L. Graduated driver licensing for reducing motor vehicle crashes among young drivers. Cochrane Database Syst Rev. (2011) 10:CD003300. 10.1002/14651858.CD003300.pub3 PMC13110912 21975738

[41] Haghpanahan H Lewsey J Mackay DF McIntosh E Pell J Jones A. An evaluation of the effects of lowering blood alcohol concentration limits for drivers on the rates of road traffic accidents and alcohol consumption: a natural experiment. Lancet. (2019) 393:321–9. 10.1016/S0140-6736(18)32850-2 30553498 PMC6346081

[42] Li L Scherpbier R Wu J Zhu X Zhang W Zhang L. Legislation coverage for child injury prevention in China. Bull World Health Organ. (2015) 93:169–75. 10.2471/BLT.14.139998 25838612 PMC4371490

[43] Ederer DJ Bui TV Parker EM Roehler DR Sidik M Florian MJ. Helmets for kids: evaluation of a school-based helmet intervention in Cambodia. Inj Prev. (2016) 22:52–8. 10.1136/injuryprev-2014-041434 26307107 PMC6883409

[44] Li C Jiao J Hua G Yundendorj G Liu S Yu H. Global burden of all cause-specific injuries among children and adolescents from 1990 to 2019: a prospective cohort study. Int J Surg. (2024) 110:2092–103. 10.1097/JS9.0000000000001131 38348839 PMC11020088

[45] van Der Lubbe SCC Moberg ME Aluquin D Zhou Y Lenox H Abdullah H. Global, regional, and national burden of road injuries 1990–2023: a systematic analysis for the global burden of disease study 2023. Lancet Public Health. (2026) 11:e589–603. 10.1016/S2468-2667(26)00141-6 42476159 PMC13487794

