# Supplementary material for: Transport-injury burden among children and adolescents in the Western Pacific Region, 2000–2023, with projections to 2035
Source: Front Pediatr. 2026 Oct 14;14:1946652. doi: 10.3389/fped.2026.1946652 (PMC13714921; doi:10.3389/fped.2026.1946652)
Supplement: Supplementary file 1 [file Supplementaryfile2.docx]

Supplementary Methods S2

**Decomposition of changes in transport-injury burden into population size, ageing, and epidemiological change**

# S2.1 Rationale and choice of method

To quantify the relative contributions of three demographic and epidemiological drivers to the change in transport-injury burden among people aged <20 years in the WHO Western Pacific Region between 2000 and 2023, we decomposed the total change in each of four burden measures—incidence, deaths, prevalence, and disability-adjusted life years (DALYs)—into three additive components: (i) population size, (ii) population ageing (age-structure change), and (iii) epidemiological change (age-specific rates). We used the Das Gupta symmetric weighted-sum (“full”) decomposition (Das Gupta, 1993). Unlike sequential (stepwise, Kitagawa-type) decompositions, the symmetric method is independent of the order in which factors are introduced, is invariant to the choice of reference population, and distributes all interaction (joint) terms equally among the three components; consequently, the three components sum exactly to the observed total change for each measure.

# S2.2 Data and notation

Age-specific population counts and age-specific estimates of incidence, mortality (deaths), prevalence, and DALYs for transport injuries were obtained from the Global Burden of Disease (GBD) 2023 study (14). GBD rates, reported per 100,000 persons, were divided by 10^5^ to express them as per-person rates. Analyses used the standard GBD five-year age groups spanning 0–19 years (<5, 5–9, 10–14, and 15–19 years) and were performed separately for both sexes combined, males, and females.

For a given burden measure, the total count in year t is expressed as the product of total population size, the age distribution of the population, and age-specific rates:

*D_t_ = Σ_i_ (p_t_ · a_it_ · r_it_) = p_t_ · Σ_i_ (a_it_ · r_it_),*

using the notation defined in Table S3.1.

**S2.3 Notation used in the decomposition analysis**

| Symbol | Description |
| --- | --- |
| i | Age-group index (i = 1, …, n) |
| t | Time point (t = 1: 2000; t = 2: 2023) |
| p_t | Total population (aged <20 years) in year t |
| a_it | Proportion of the population in age group i in year t (Σ_i a_it = 1) |
| r_it | Age-specific rate (per person) in age group i and year t for the measure under analysis (incidence, death, prevalence, or DALY rate; GBD rate per 100,000 ÷ 10^{5}) |
| D_t | Total burden count in year t (incident cases, deaths, prevalent cases, or DALYs) |
| ΔD | Change in the total burden count between 2000 and 2023 (D_2 − D_1) |
| ΔD_pop | Population-size component of the change |
| ΔD_age | Population-ageing (age-structure) component of the change |
| ΔD_rate | Epidemiological-change (rate) component of the change |

# S2.4 Decomposition

Let ΔD = D_2 − D_1. The Das Gupta decomposition partitions ΔD into three additive components (19):

*ΔD = ΔD_pop_ + ΔD_age_ + ΔD_rate_.*

The three components are defined by the closed-form expressions below. The 1/3 and 1/6 coefficients weight each factor’s contribution over the four possible combinations of the remaining two factors’ start/end values, which is the defining property of the symmetric method.

**(i) Population-size effect:**

*ΔD_pop_ = (p_2_ − p_1_) × [ (1/3)Σ_i_ a_i1_ r_i1_ + (1/6)Σ_i_ a_i1_ r_i2_ + (1/6)Σ_i_ a_i2_ r_i1_ + (1/3)Σ_i_ a_i2_ r_i2_ ]*

**(ii) Population-ageing effect:**

*ΔD_age_ = Σ_i_ (a_i2_ − a_i1_) × [ (1/3)p_1_ r_i1_ + (1/6)p_1_ r_i2_ + (1/6)p_2_ r_i1_ + (1/3)p_2_ r_i2_ ]*

**(iii) Epidemiological-change effect:**

*ΔD_rate_ = Σ_i_ (r_i2_ − r_i1_) × [ (1/3)p_1_ a_i1_ + (1/6)p_1_ a_i2_ + (1/6)p_2_ a_i1_ + (1/3)p_2_ a_i2_ ]*

By construction the three terms are mutually exclusive and exhaustive: ΔD_pop_ + ΔD_age_ + ΔD_rate_ ≡ ΔD, with all interaction effects allocated symmetrically across the three components.

# S2.5 Implementation and validation

All computations were performed in R (version 4.5.2) using the dplyr, data.table, tidyr, and purrr packages. For each burden measure and sex stratum, the three components were computed directly from the closed-form expressions in Section S3.3; the corresponding R scripts are available from the authors on request. The additivity condition (sum of components equals the observed total change) was verified for every analysis; residual differences, attributable solely to floating-point precision, were <1 × 10^−9^.

# S2.6 Percentage contributions

Percentage contributions were obtained by dividing each component by the total change:

*%Population = (ΔD_pop_ / ΔD) × 100; %Ageing = (ΔD_age_ / ΔD) × 100; %Epidemiological = (ΔD_rate_ / ΔD) × 100.*

The three percentages sum to 100%. Where the total change was small or components carried opposite signs, percentages were interpreted with caution and reported alongside the absolute values.

# References

19. Das Gupta P. Standardization and Decomposition of Rates: A User’s Manual. Current Population Reports, Series P23-186. Washington, DC: U.S. Bureau of the Census, U.S. Government Printing Office; 1993.

14. GBD 2023 Diseases and Injuries Collaborators. Global burden of 375 diseases and injuries, risk-attributable burden of 88 risk factors, and healthy life expectancy in 204 countries and territories, including 660 subnational locations, 1990–2023: a systematic analysis for the Global Burden of Disease Study 2023. Lancet. 2025; published online Oct 12. doi:10.1016/S0140-6736(25)01637-X.
