# Supplementary material for: Transport-injury burden among children and adolescents in the Western Pacific Region, 2000–2023, with projections to 2035
Source: Front Pediatr. 2026 Oct 14;14:1946652. doi: 10.3389/fped.2026.1946652 (PMC13714921; doi:10.3389/fped.2026.1946652)
Supplement: Supplementary file 2 [file Supplementaryfile3.docx]

# Supplementary Methods 3 — ARIMA forecasting details

## Stationarity

The stationarity of each sex- and indicator-specific series was assessed using the augmented Dickey–Fuller (ADF) test; first-order differencing (d = 1) was applied to render each series stationary.

## Model specification

Following Behera et al. (20), each series was modeled as ARIMA (1,1,1), where the first-order autoregressive term (AR,1) accounts for the relationship between the previous and current values of the series, first-order differencing (I,1) renders the series stationary, and the first-order moving-average term (MA,1) captures random noise and shocks in the data (21).

## Modeling unit

Models were fitted separately by sex for each of the four indicators on the study-level age-standardized rate series (a single global series per sex–indicator combination).

## Forecasting horizon and non-negativity

Forecasts and 95% prediction intervals were generated from the fitted models. Because ARIMA predictions are unconstrained, strong downward trends can yield zero or negative forecasts that are epidemiologically implausible. The projection horizon was therefore restricted to 2024–2035, within which all point forecasts and 95% prediction intervals remained strictly positive for every indicator.

**Sensitivity analysis**

As a sensitivity analysis, each sex- and indicator-specific series was additionally modelled using log-transformed ARIMA (1,1,1), which enforces strictly positive forecasts on back-transformation and imposes a multiplicative rather than additive decline. Within the 2024–2035 projection horizon, the log-transformed models yielded trajectories consistent with the primary models for incidence and prevalence (within ±14% at 2035), but produced substantially more conservative forecasts for mortality and DALYs (143–279% higher at 2035; Supplementary Tables C3 and C4; Fig. S1). These differences indicate that the near-zero mortality and DALY projections are sensitive to the assumption of an additive linear decline and should be interpreted with caution.

## Software

Analyses were performed in R (version 4.5.2); P < 0.05 was considered statistically significant.

## ARIMA model specification and stationarity assessment for each sex- and indicator-specific series

| **Sex** | **Indicator** | **ARIMA (p, d, q)** | **Model specification** | **Differencing (d)** | **Stationarity test** |
| --- | --- | --- | --- | --- | --- |
| Male | Incidence | (1, 1, 1) | ARIMA (1,1,1) | 1 | ADF test |
| Male | Prevalence | (1, 1, 1) | ARIMA (1,1,1) | 1 | ADF test |
| Male | Mortality | (1, 1, 1) | ARIMA (1,1,1) | 1 | ADF test |
| Male | DALYs | (1, 1, 1) | ARIMA (1,1,1) | 1 | ADF test |
| Female | Incidence | (1, 1, 1) | ARIMA (1,1,1) | 1 | ADF test |
| Female | Prevalence | (1, 1, 1) | ARIMA (1,1,1) | 1 | ADF test |
| Female | Mortality | (1, 1, 1) | ARIMA (1,1,1) | 1 | ADF test |
| Female | DALYs | (1, 1, 1) | ARIMA (1,1,1) | 1 | ADF test |

ARIMA, autoregressive integrated moving average; ADF, augmented Dickey–Fuller. A parsimonious fixed-order ARIMA (1,1,1) specification was adopted for all series, consistent with Behera et al. [1]. First-order differencing (d = 1) rendered all series stationary.

# References

20. Behera DK, Rahut DB, Tripathy S. Alzheimer's disease and dementia in Japan: Epidemiological trends, regional disparities, and future projections. Alzheimers Dement. 2025 Jul;21(7):e70444. doi: 10.1002/alz.70444.

21. Duo T, Wen Y, Bian Y, Wang Y, Zhang X, Ju J, Lu Y, Wang Z, Wang J, Yang B. Rising burden of MASLD and CKM syndrome in Asia: A decade of trends and future projections. Metabolism. 2026 May;178:156549. doi: 10.1016/j.metabol.2026.156549. Epub 2026 Feb 3.
