# Supplementary material for: Transport-injury burden among children and adolescents in the Western Pacific Region, 2000–2023, with projections to 2035
Source: Front Pediatr. 2026 Oct 14;14:1946652. doi: 10.3389/fped.2026.1946652 (PMC13714921; doi:10.3389/fped.2026.1946652)
Supplement: Supplementary file 3 [file Table1.docx]

Supplementary Table A Geographic segmentation of incidence, prevalence, and death in the Western Pacific

| Location name | Incidence number | ASIR | Prevalence number | ASPR | Death number | ASMR |
| --- | --- | --- | --- | --- | --- | --- |
| Western Pacific Region | 2433401.58 | 428.74 | 1468431.49 | 247.96 | 28378.99 | 4.94 |
| Tuvalu | 13.09 | 301.99 | 7.36 | 169.85 | 0.69 | 15.72 |
| Tokelau | 1.25 | 216.97 | 0.85 | 137.71 | 0.02 | 3.63 |
| Palau | 15.15 | 338.16 | 8.87 | 193.34 | 0.4 | 8.79 |
| Northern Mariana Islands | 44.04 | 313.05 | 30.19 | 196.63 | 0.81 | 5.32 |
| Niue | 1.78 | 344.08 | 1.12 | 207.38 | 0.02 | 3.11 |
| Nauru | 28.56 | 502.95 | 16.54 | 292.58 | 1.32 | 23.28 |
| Guam | 136.03 | 285.97 | 84.33 | 173.51 | 1.45 | 2.97 |
| Cook Islands | 13.39 | 262.48 | 7.92 | 150.44 | 0.41 | 7.9 |
| American Samoa | 41.21 | 219.59 | 27.66 | 138.58 | 0.67 | 3.36 |
| New Zealand | 10795.66 | 793.4 | 4986.68 | 358.74 | 53.13 | 3.85 |
| Australia | 39066.05 | 588.7 | 19050.4 | 280.56 | 162.14 | 2.4 |
| Singapore | 6166.02 | 616.05 | 3628.41 | 357.83 | 6.01 | 0.61 |
| Republic of Korea | 56093.1 | 716.04 | 35395.39 | 404.4 | 147.83 | 1.72 |
| Japan | 105197.51 | 504.69 | 60402.55 | 273.21 | 161.74 | 0.73 |
| Brunei Darussalam | 1252.48 | 971.55 | 678.33 | 506.81 | 3.36 | 2.57 |
| Mongolia | 6308.29 | 465.9 | 3761.22 | 286.93 | 68.76 | 5.12 |
| Vanuatu | 549.42 | 365.94 | 311.56 | 210.13 | 2.36 | 1.58 |
| Tonga | 111.81 | 236.12 | 72.27 | 147.51 | 1.92 | 4.01 |
| Solomon Islands | 1156.67 | 285.75 | 616.12 | 153.07 | 7.7 | 1.91 |
| Samoa | 286.41 | 283.65 | 164.05 | 163.56 | 5.16 | 5.02 |
| Papua New Guinea | 25974.18 | 492.72 | 16136.76 | 323.46 | 449.44 | 9.07 |
| Micronesia (Federated States of) | 139.12 | 327.82 | 81.82 | 188.65 | 1.75 | 4.07 |
| Marshall Islands | 53.56 | 289.93 | 33.52 | 177.69 | 0.69 | 3.64 |
| Kiribati | 151.07 | 275.1 | 93.78 | 171.06 | 2.62 | 4.75 |
| Fiji | 986.45 | 278.65 | 657.35 | 182.16 | 18.03 | 5.06 |
| Viet Nam | 129628.44 | 406.2 | 62022 | 191.14 | 2128.29 | 6.58 |
| Philippines | 111247.8 | 248.37 | 71392.24 | 153.16 | 2177.09 | 4.77 |
| Malaysia | 44370.22 | 395.42 | 20113.55 | 177.8 | 686.93 | 5.96 |
| Lao People's Democratic Republic | 10295.48 | 345.42 | 5810.41 | 195.23 | 290.15 | 9.7 |
| Indonesia | 356182.91 | 387.66 | 218106.07 | 231.92 | 10228.98 | 10.72 |
| Cambodia | 23206.53 | 350.95 | 12258.67 | 185.17 | 699.57 | 10.56 |
| Taiwan (Province of China) | 17539.16 | 431.56 | 8616.3 | 208.33 | 174.95 | 4.14 |
| China | 1504110.41 | 452.29 | 932616.52 | 266.03 | 11072.53 | 3.41 |

ASIR, age-standardized incidence rate; ASPR, Age-Standardized Prevalence Rate; ASMR, Age-Standardized Mortality Rate
