# Supplementary material for: Transport-injury burden among children and adolescents in the Western Pacific Region, 2000–2023, with projections to 2035
Source: Front Pediatr. 2026 Oct 14;14:1946652. doi: 10.3389/fped.2026.1946652 (PMC13714921; doi:10.3389/fped.2026.1946652)
Supplement: Supplementary file 4 [file Table2.docx]

**Supplementary Table-B1 Male and age strata for incidence and prevalence of transport injuries, 2000-2023**

| **Years/** | **No.% (95% UI)** | | **No. (95% UI)** | |
| --- | --- | --- | --- | --- |
| **Male** | **Number of Incidence Rate of Incidence** | | **Number of Prevalence Rate of Prevalence** | |
| <5 | 234947.67 (194798.09, 281318.30) | 383.29 (317.79, 458.94) | 58695.37 (52416.89, 65292.34) | 95.75 (85.51, 106.52) |
| 5-9 | 254659.28 (179030.26, 338572.81) | 302.82 (212.89, 402.60) | 143770.87 (126246.54, 161278.91) | 170.96 (150.12, 191.78) |
| 10-14 | 298699.54 (204094.86, 421115.49) | 382.62 (261.44, 539.43) | 218198.37 (196514.04, 247126.55) | 279.50 (251.72, 316.56) |
| 15-19 | 726215.44 (523631.51, 976507.17) | 996.81 (718.74, 1340.36) | 405670.64 (364388.61, 455804.98) | 556.83 (500.16, 625.64) |

**Supplementary Table-B2 Female and age strata for incidence and prevalence of transport injuries, 2000-2023**

| **Years/** | **No.% (95% UI)** | | **No. (95% UI)** | |
| --- | --- | --- | --- | --- |
| **Female** | **Number of Incidence Rate of Incidence** | | **Number of Prevalence Rate of Prevalence** | |
| <5 | 222698.42 (184317.91, 265904.32) | 402.33 (332.99, 480.38) | 57758.33 (51683.54, 63987.04) | 104.35 (93.37, 115.60) |
| 5-9 | 203423.65 (139608.84, 276508.28) | 267.91 (183.87, 364.16) | 133119.81 (116749.16, 150437.13) | 175.32 (153.76, 198.13) |
| 10-14 | 207998.88 (138426.71, 300421.21) | 294.79 (196.19, 425.78) | 186502.62 (167256.91, 208701.45) | 264.33 (237.05, 295.79) |
| 15-19 | 284758.70 (202632.06, 378681.98) | 434.88 (309.46, 578.32) | 264715.47 (238557.36, 300046.06) | 404.27 (364.32, 458.23) |

**Supplementary Table-B3 Male and age strata for death and DALY of transport injuries, 2000-2023**

| **Years/** | **No.% (95% UI)** | | **No. (95% UI)** | |
| --- | --- | --- | --- | --- |
| **Male** | **Number of Deaths Rate of Deaths** | | **Number of DALYs Rate of DALYs** | |
| <5 | 2699.04 (1578.09, 3607.78) | 4.40 (2.57, 5.89) | 240023.32 (141674.27, 319376.06) | 391.57 (231.13, 521.03) |
| 5-9 | 2744.45 (1776.57, 3770.31) | 3.26 (2.11, 4.48) | 235327.59 (154382.15, 319917.98) | 279.83 (183.58, 380.42) |
| 10-14 | 3447.36 (2141.85, 4496.80) | 4.42 (2.74, 5.76) | 279594.36 (178248.31, 362568.12) | 358.15 (228.33, 464.43) |
| 15-19 | 10396.97 (7615.57, 13190.69) | 14.27 (10.45, 18.11) | 778258.99 (577522.42, 981898.25) | 1068.25 (792.71, 1347.76) |

**Supplementary Table-B4 Female and age strata for death and DALY of transport injuries, 2000-2023**

| **Years/** | **No.% (95% UI)** | | **No. (95% UI)** | |
| --- | --- | --- | --- | --- |
| **Female** | **Number of Deaths Rate of Deaths** | | **Number of DALYs Rate of DALYs** | |
| <5 | 1819.46 (1084.71, 2502.54) | 3.29 (1.96, 4.52) | 162713.63 (97986.47, 222635.36) | 293.96 (177.02, 402.21) |
| 5-9 | 1798.95 (1154.24, 2451.07) | 2.37 (1.52, 3.23) | 156058.87 (102352.80, 209967.17) | 205.53 (134.80, 276.53) |
| 10-14 | 1768.63 (1135.19, 2432.68) | 2.51 (1.61, 3.45) | 147106.38 (98735.77, 198091.76) | 208.49 (139.94, 280.75) |
| 15-19 | 3704.12 (2277.49, 4951.66) | 5.66 (3.48, 7.56) | 283010.81 (178903.04, 375019.27) | 432.21 (273.22, 572.73) |

UI, uncertainty interval; DALYs, Disability-Adjusted Life Years
