# Supplementary material for: Transport-injury burden among children and adolescents in the Western Pacific Region, 2000–2023, with projections to 2035
Source: Front Pediatr. 2026 Oct 14;14:1946652. doi: 10.3389/fped.2026.1946652 (PMC13714921; doi:10.3389/fped.2026.1946652)
Supplement: Supplementary file 5 [file Table3.docx]

**Supplementary Table C1 – Male-Future prediction analysis of incidence, prevalence, death and DALY of transport injuries,2024-2035**.

| **Years/Male** | **Incidence Value (95% CI)** | **Prevalence Value (95% CI)** | **Death Value (95% CI)** | **DALYS Value (95% CI)** |
| --- | --- | --- | --- | --- |
| 2024 | 497.96(486.32,515.38) | 262.45(254.57,272.25) | 5.89(5.67,6.20) | 471.41(453.91,496.30) |
| 2025 | 495.79(480.05,521.57) | 260.72(248.45,275.24) | 5.47(5.29,5.78) | 437.70(423.82,462.98) |
| 2026 | 492.38(470.31,524.81) | 258.49(242.95,277.73) | 5.03(4.81,5.36) | 402.95(386.80,429.64) |
| 2027 | 489.78(463.38,530.18) | 256.59(236.82,280.49) | 4.61(4.40,4.94) | 368.82(352.36,396.88) |
| 2028 | 486.62(454.30,533.97) | 254.47(231.00,283.14) | 4.18(3.94,4.53) | 334.23(316.36,363.75) |
| 2029 | 483.87(447.50,539.27) | 252.51(225.29,285.56) | 3.75(3.50,4.12) | 299.97(280.90,330.71) |
| 2030 | 480.79(438.40,543.81) | 250.42(219.50,288.06) | 3.32(3.06,3.71) | 265.43(245.20,297.53) |
| 2031 | 477.98(431.18,548.70) | 248.43(213.64,290.62) | 2.89(2.61,3.30) | 231.11(209.48,264.43) |
| 2032 | 474.96(422.23,553.30) | 246.37(207.78,293.01) | 2.46(2.17,2.88) | 196.61(174.00,231.26) |
| 2033 | 472.11(414.79,558.09) | 244.36(201.85,295.39) | 2.03(1.72,2.47) | 162.25(138.62,198.09) |
| 2034 | 469.12(406.24,562.76) | 242.31(195.97,297.77) | 1.60(1.27,2.06) | 127.77(103.23,164.92) |
| 2035 | 466.24(398.68,567.41) | 240.29(189.99,300.15) | 1.17(0.83,1.65) | 93.39(67.84,131.75) |

DALYs, Disability-Adjusted Life Years; CI, confidence interval.

**Supplementary Table C2 – Female-Future prediction analysis of incidence, prevalence, death and DALY of transport injuries,2024-2035**.

| **Years/Female** | **Incidence Value (95% CI)** | **Prevalence Value (95% CI)** | **Death Value (95% CI)** | **DALYS Value (95% CI)** |
| --- | --- | --- | --- | --- |
| 2024 | 349.33(335.21,360.68) | 229.85(219.04,236.65) | 3.13(2.96,3.33) | 258.11(245.01,271.82) |
| 2025 | 349.56(329.99,367.35) | 229.61(213.39,239.87) | 2.94(2.76,3.13) | 241.84(228.55,255.30) |
| 2026 | 349.62(324.91,373.56) | 229.34(207.95,244.32) | 2.72(2.54,2.94) | 223.09(210.10,237.80) |
| 2027 | 349.79(319.30,379.87) | 229.08(202.29,248.58) | 2.51(2.32,2.74) | 205.90(191.93,221.05) |
| 2028 | 349.90(313.57,386.79) | 228.81(196.68,252.91) | 2.30(2.11,2.53) | 187.44(173.20,203.56) |
| 2029 | 350.05(307.36,393.70) | 228.57(191.22,257.12) | 2.09(1.89,2.33) | 169.93(155.13,187.23) |
| 2030 | 350.17(301.84,400.62) | 228.30(185.66,261.03) | 1.88(1.68,2.13) | 151.85(136.60,170.08) |
| 2031 | 350.31(295.78,407.54) | 228.05(180.18,264.97) | 1.67(1.46,1.93) | 134.07(118.33,153.58) |
| 2032 | 350.43(290.10,414.46) | 227.79(174.69,269.07) | 1.46(1.24,1.73) | 116.09(99.79,136.21) |
| 2033 | 350.58(284.16,421.37) | 227.53(169.21,273.25) | 1.25(1.03,1.53) | 98.30(81.37,119.37) |
| 2034 | 350.69(278.40,428.29) | 227.27(163.73,277.44) | 1.04(0.81,1.33) | 80.34(62.82,102.34) |
| 2035 | 350.83(272.52,435.21) | 227.02(158.25,281.62) | 0.83(0.59,1.13) | 62.50(44.26,85.41) |

DALYs, Disability-Adjusted Life Years; CI, confidence interval.

**Supplementary Table C3 log – Male-Future prediction analysis of incidence, prevalence, death and DALY of transport injuries (log-transformed ARIMA (1,1,1) sensitivity analysis), 2024–2035.**

| **Years/Male** | **Incidence Value**  **(95% CI)** | **Prevalence Value (95% CI)** | **Death Value (95% CI)** | **DALYS Value**  **(95% CI)** |
| --- | --- | --- | --- | --- |
| 2024 | 504.15(496.90,511.51) | 265.28(261.17,269.45) | 6.11(5.92,6.31) | 490.20(473.89,507.08) |
| 2025 | 507.20(490.01,525.00) | 266.10(256.21,276.37) | 5.89(5.57,6.23) | 474.04(446.50,503.28) |
| 2026 | 510.09(481.17,540.75) | 266.89(250.17,284.72) | 5.68(5.23,6.17) | 458.80(420.17,500.99) |
| 2027 | 512.81(470.98,558.36) | 267.64(243.41,294.27) | 5.49(4.91,6.14) | 444.43(394.82,500.27) |
| 2028 | 515.38(459.85,577.62) | 268.35(236.17,304.91) | 5.30(4.60,6.11) | 430.86(370.52,501.02) |
| 2029 | 517.81(448.09,598.38) | 269.02(228.62,316.56) | 5.13(4.31,6.11) | 418.03(347.33,503.12) |
| 2030 | 520.10(435.93,620.53) | 269.67(220.91,329.19) | 4.96(4.03,6.12) | 405.90(325.29,506.47) |
| 2031 | 522.27(423.55,643.99) | 270.28(213.13,342.75) | 4.81(3.77,6.14) | 394.41(304.42,511.01) |
| 2032 | 524.31(411.08,668.72) | 270.86(205.37,357.23) | 4.66(3.52,6.17) | 383.54(284.70,516.68) |
| 2033 | 526.23(398.64,694.66) | 271.41(197.68,372.64) | 4.52(3.28,6.22) | 373.23(266.12,523.43) |
| 2034 | 528.04(386.30,721.79) | 271.93(190.12,388.95) | 4.39(3.06,6.28) | 363.45(248.65,531.23) |
| 2035 | 529.75(374.15,750.07) | 272.43(182.73,406.18) | 4.26(2.86,6.35) | 354.16(232.25,540.06) |

Values are age-standardised rates per 100,000 population, presented as point forecast (95% prediction interval). Log-transformed ARIMA (1,1,1) forecasts enforce positivity on back-transformation. DALYs, Disability-Adjusted Life Years; CI, confidence interval.

**Supplementary Table C4 log – Female-Future prediction analysis of incidence, prevalence, death and DALY of transport injuries (log-transformed ARIMA (1,1,1) sensitivity analysis), 2024–2035.**

| **Years/Female** | **Incidence Value**  **(95% CI)** | **Prevalence Value (95% CI)** | **Death Value (95% CI)** | **DALYS Value**  **(95% CI)** |
| --- | --- | --- | --- | --- |
| 2024 | 349.54(344.16,355.01) | 229.38(225.67,233.14) | 3.24(3.10,3.40) | 269.16(257.13,281.76) |
| 2025 | 350.54(337.24,364.36) | 229.10(220.06,238.52) | 3.10(2.89,3.33) | 257.54(240.13,276.22) |
| 2026 | 351.49(328.82,375.72) | 228.84(213.49,245.28) | 2.97(2.70,3.26) | 246.47(224.72,270.32) |
| 2027 | 352.39(319.40,388.79) | 228.59(206.32,253.25) | 2.84(2.53,3.19) | 235.92(210.42,264.52) |
| 2028 | 353.25(309.32,403.42) | 228.34(198.77,262.32) | 2.72(2.36,3.12) | 225.87(197.03,258.93) |
| 2029 | 354.07(298.84,419.51) | 228.11(191.00,272.43) | 2.60(2.21,3.06) | 216.29(184.45,253.62) |
| 2030 | 354.85(288.15,437.00) | 227.89(183.15,283.56) | 2.49(2.07,3.00) | 207.15(172.62,248.59) |
| 2031 | 355.59(277.38,455.86) | 227.68(175.31,295.69) | 2.38(1.93,2.94) | 198.44(161.49,243.86) |
| 2032 | 356.30(266.66,476.08) | 227.48(167.55,308.83) | 2.28(1.80,2.89) | 190.13(151.01,239.40) |
| 2033 | 356.97(256.06,497.65) | 227.28(159.94,322.99) | 2.19(1.68,2.84) | 182.21(141.15,235.21) |
| 2034 | 357.61(245.66,520.58) | 227.10(152.50,338.19) | 2.10(1.57,2.80) | 174.65(131.88,231.29) |
| 2035 | 358.22(235.50,544.87) | 226.92(145.28,354.44) | 2.01(1.46,2.76) | 167.43(123.17,227.61) |

Values are age-standardised rates per 100,000 population, presented as point forecast (95% prediction interval). Log-transformed ARIMA (1,1,1) forecasts enforce positivity on back-transformation. DALYs, Disability-Adjusted Life Years; CI, confidence interval.
